# Nanovector-based prolyl hydroxylase domain 2 silencing system enhances the efficiency of stem cell transplantation for infarcted myocardium repair

**DOI:** 10.2147/IJN.S71586

**Published:** 2014-11-11

**Authors:** Kai Zhu, Hao Lai, Changfa Guo, Jun Li, Yulin Wang, Lingyan Wang, Chunsheng Wang

**Affiliations:** 1 Department of Cardiac Surgery, Zhongshan Hospital, Fudan University, Shanghai, People’s Republic of China; 2 Shanghai Institute of Cardiovascular Disease, Shanghai, People’s Republic of China; 3 Biomedical Research Center, Zhongshan Hospital, Fudan University, Shanghai, People’s Republic of China

**Keywords:** nanoparticles, PHD2, siRNA delivery, mesenchymal stem cells, myocardial infarction

## Abstract

Mesenchymal stem cell (MSC) transplantation has attracted much attention in myocardial infarction therapy. One of the limitations is the poor survival of grafted cells in the ischemic microenvironment. Small interfering RNA-mediated prolyl hydroxylase domain protein 2 (PHD2) silencing in MSCs holds tremendous potential to enhance their survival and paracrine effect after transplantation. However, an efficient and biocompatible PHD2 silencing system for clinical application is lacking. Herein, we developed a novel PHD2 silencing system based on arginine-terminated generation 4 poly(amidoamine) (Arg-G4) nanoparticles. The system exhibited effective and biocompatible small interfering RNA delivery and PHD2 silencing in MSCs in vitro. After genetically modified MSC transplantation in myocardial infarction models, MSC survival and paracrine function of IGF-1 were enhanced significantly in vivo. As a result, we observed decreased cardiomyocyte apoptosis, scar size, and interstitial fibrosis, and increased angiogenesis in the diseased myocardium, which ultimately attenuated ventricular remodeling and improved heart function. This work demonstrated that an Arg-G4 nanovector-based PHD2 silencing system could enhance the efficiency of MSC transplantation for infarcted myocardium repair.

## Introduction

Mesenchymal stem cell (MSC) transplantation is a promising therapy strategy for cardiac repair in myocardial infarction (MI). It appears that grafted MSCs can salvage myocardium from death through a protective paracrine mechanism.^1^,^2^ However, one limitation of the therapy is that grafted cells suffer from oxidative injury in the harsh ischemic microenvironment and can hardly survive after transplantation. How to improve the survival of grafted MSCs is a demanding task for further research.^3^–^5^ One strategy is to manipulate the expression of key genes involved in the cell survival. In the previous studies, genetic modification of MSCs overexpressing some pro-survival genes, such as *Akt, Bcl-2, stromal-derived factor 1* (*SDF-1*), and *chemokine (c-c motif) receptor-1*, had been tested to improve the survival of grafted cells in vivo but with little efficacy.^3^,^6^ Small interfering RNA (siRNA)-mediated silencing of oxidative injury-related gene expression may open a novel possibility for enhancing cell survival. As an oxygen-dependent gene, the *prolyl hydroxylase domain protein 2* (*PHD2*) gene regulates hypoxia-inducible factor-1α (HIF-1α) and nuclear factor-κB (NF-κB), which are involved in cell survival and inflammation.^7^,^8^ Recently, it has been demonstrated that lentivirus-based PHD2 silencing in stem cells could enhance the survival of grafted cells in ischemic tissue significantly through a HIF-1α dependent pathway and secretion of insulin-like growth factor-1 (IGF-1) via an NF-κB-mediated mechanism.^9^–^11^ However, for clinical application, an effective and safe non-viral PHD-2 silencing system remains in great demand.

Currently, non-viral vector-based siRNA delivery strategies has been of great interest, since they have the potential to be safer and less toxic than viral vectors. Cationic polymers, lipid carriers, supramolecular nanocarriers, and other non-viral vectors have been applied for intracellular delivery of siRNA.^12^–^14^ Among these, poly(amidoamine) (PAMAM) dendrimers represent a promising siRNA delivery vector by virtue of their precisely controlled radial symmetrical structure, intriguing multivalency, and high cargo payload confined within a nanoscale volume. The positive charges born on their terminal amine groups result in high buffering capacity in cells, which promotes the endosomal escape and siRNA transfection.^15^,^16^ Though high generations of PAMAM exhibit more excellent gene/siRNA delivery and cell-penetrating abilities than lower ones, the large-scale synthesis of high generations is technically demanding and economically costly. To achieve the economical and effective delivery, terminal-modification on lower generations of PAMAM is worthwhile for further application.^17^,^18^ It has been proved that arginine-rich peptides, such as cell-penetration peptides, transactivator of transcription, penetratin, and oligoarginine, can promote cell membrane penetration with their double positively charged arginine residues. Therefore, low generations of PAMAM had been conjugated with arginine residues to empower their drug/gene/siRNA delivery abilities.^18^–^20^

**Figure 1:**
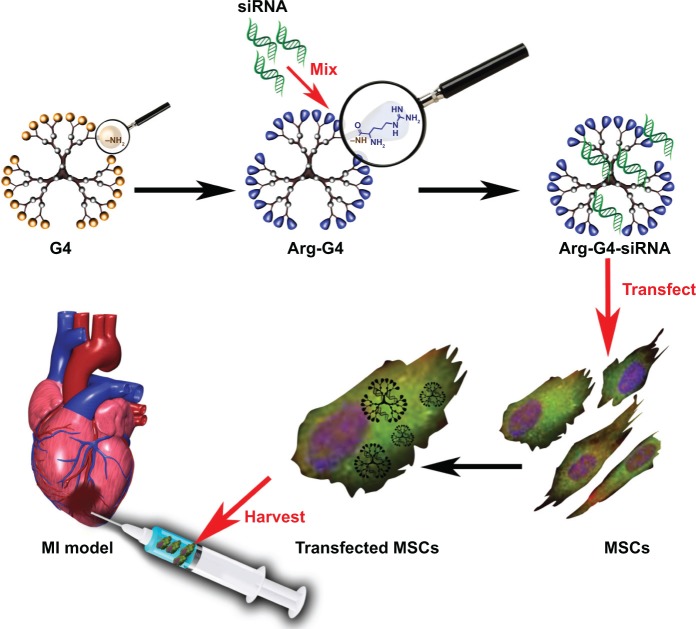
Schematic representation of Arg-G4-based PHD2 silencing system combined with MSC transplantation for infarcted myocardium repair. **Abbreviations:** Arg-G4, arginine-terminated G4; G4, generation 4 poly(amidoamine); MI, myocardial infarction; MSC, mesenchymal stem cell; PHD2, prolyl hydroxylase domain protein 2; siRNA, small interfering RNA.

Our present study developed an arginine-terminated generation 4 PAMAM (Arg-G4) nanoparticle-based PHD2 silencing system. We hypothesized that this novel PHD2 silencing system could enable an effective and biocompatible strategy to enhance MSC survival and paracrine function after transplantation, and promote repair of the ischemic myocardium. The schematic representation of the Arg-G4-based therapy strategy is shown in Figure 1.

## Materials and methods

### Preparation of Arg-G4 nanoparticles

Arg-G4 nanoparticles were synthesized as previously described, with some modification.^19^ Briefly, 50 mg of generation 4 PAMAM (G4) (Sigma-Aldrich Co., St Louis, MO, USA) was dissolved in 5 mL of anhydrous N,N-dimethylformamide (DMF; Sigma-Aldrich Co.) as solution A. Four equivalents of 1-hydroxybenzotriazole (Anaspec, San Jose, CA, USA), four equivalents of O-(benzotriazol-1-yl)-N,N,N′,N′-tetramethyluronium hexafluorophosphate (Anaspec), four equivalents of Fmoc-Arg(pbf)-OH (Novabiochem, San Diego, CA, USA), and eight equivalents of N,N-diisopropylethylamine (Sigma-Aldrich Co.) were dissolved in 5 mL of DMF as solution B. Solution A was added into solution B at room temperature. The mixture was then stirred for reaction at 25°C for 48 hours. Afterward, the product was precipitated with ethyl ether at 4°C for 12 hours, which was performed three times in succession. The residual precipitate was dissolved in 3.0 mL of DMF and mixed with a solution of 3.0 mL of 30% piperidine in DMF (v/v). The resulting solution was then stirred at 25°C for 2 hours to deprotect Fmoc groups. After the precipitation process as above, the resulting precipitate was washed with excess ether. To remove the pbf groups, a solution of 7.6 mL of trifluoroacetic acid, 2.0 mL of triisopropylsilane, and 2.0 mL of water (95:2.5:2.5, v/v/v) was added into the resulting precipitate at 25°C for 1 hour. The product was solubilized in water and dialyzed against pure water at 4°C overnight. The dialyzed product was lyophilized and freeze-dried to yield the powder of Arg-G4 nanoparticles.

### Preparation of Arg-G4-siRNA complexes

Arg-G4-siRNA was prepared by the simple commixture. Arg-G4 nanoparticles were dissolved in 50 mM Tris-HCl buffer (pH 7.4). PHD2 siRNA (Santa Cruz Biotechnology, Santa Cruz, Dallas, TX, USA), which was dissolved in DEPC (diethylpyrocarbonate) water, was dropwise added into the nanoparticle solution at various nanoparticle to DNA nitrogen-phosphorus (N/P) ratios (1, 5, 10, 15, and 20). At room temperature, the mixture was immediately vortexed gently for 20 minutes and then allowed to sediment for 10 minutes. The resulting Arg-G4-siRNA complexes were used for the following experiments freshly.

### Characterizations of Arg-G4-siRNA complexes

The shape of the complexes was visualized using transmission electron microscopy (JEM-1230; JEOL, Tokyo, Japan). The zeta potential and particle size of the complexes were demonstrated by Zetasizer instrument (Zetasizer Nano ZS; Malvern Instruments, Malvern, UK).

To determine the resistance of Arg-G4-siRNA to degradation of RNase A (Sigma-Aldrich Co.), 1 μL of RNase A (30 ng/μL) was added into the complexes solution and incubated at 37°C for 0, 0.5, 1, 2, and 3 hours, respectively. To stop the degradation reaction, 1 μL of RNase A inhibitor (50 U/μL) was added into the solution at every time point. And then 3 μL of sodium dodecyl sulfate (10%, w/v) was added to dissociate the siRNA from the complexes. Electrophoresis was carried out on 3.5% (w/v in 1× TBE [Tris-borate-EDTA]) agarose gel. The results were analyzed to identify the remains of siRNA after the degradation of RNase A.

### Isolation and culture of primary MSCs

All animals were cared for and maintained at the Animal Center, Shanghai Medical College of Fudan University, and were approved by the Institutional Review Board and Institutional Animal Care and Use Committee Protocols of Fudan University.

Isolation of primary MSCs was performed from the bone marrow of 8-week-old male C57/BL6 mice, as described in our earlier study.^21^ Briefly, bone marrow was flushed from tibias and femurs of donor mouse. The marrow-derived cells were cultured in MesenCult basal medium (Cyagen Biosciences Inc., Goleta, CA, USA). The non-adherent cells were discharged after 36 and 72 hours, and adherent cells were cultured until confluent. All experiments were carried out with cells passaged no more than five times.

### Optimization of Arg-G4-based gene silencing system

PHD2 siRNA was tagged with carboxyfluorescein (FAM) (Sigma-Aldrich Co.) on the 5′ sense strand before cell transfection.^22^ MSCs were seeded onto 6-well plates and incubated to reach 80%–90% confluence. The culture media were then replaced with solution of Arg-G4-siRNA complexes at various N/P ratios (1, 5, 10, 15, and 20) using 50 nM of siRNA. Lipofectamine 2000 (Invitrogen, Thermo Fisher Scientific, Waltham, MA, USA)-based siRNA transfection was performed as a positive control according to the manufacture’s protocol. After incubation at 37°C for 6 hours, intracellular siRNA was observed directly under a fluorescence microscope (Olympus Corporation, Tokyo, Japan). And then, cells were harvested and collected in phosphate buffered saline (PBS) to measure the fluorescence intensity. The fluorescence intensity at 490/530 nm of FAM per well was determined as transfection efficiency by flow cytometer (FACSAriaII; BD, Franklin Lakes, NJ, USA).

Cytotoxicity of the complexes was assessed using 3-(4,5-dimethylthiazol-2-yl)-2,5-diphenyl tetrazolium bromide (MTT) assay. MSCs at the density of 8×10^3^ cells/well were seeded into 96-well plates. After 24 hours, the culture media were replaced, respectively, with 200 μL of the culture media containing Arg-G4-siRNA complexes at various N/P ratios and additionally incubated for 6 hours. Then the media were exchanged for fresh media and allowed to continue incubating. After 48 hours of incubation, 20 μL of MTT (5 mg/mL) solution was added per well and cultured for 4 hours, and then the supernatant was discarded and 100 μL of dimethylsulfoxide was added to each well. The untreated MSCs were taken as a control with 100% viability, and cells without addition of MTT were used as blank to calibrate the spectrophotometer to zero absorbance. The suspension was placed on microvibrator for 5 minutes, and the optical density was measured at 570 nm by a microplate reader (Thermo Scientific, Waltham, MA, USA).

To determine the optimized siRNA concentration, 10, 20, 50, 100, and 200 nM of siRNA were tested for transfection.

### MSC bio-behaviors during optimized gene silencing

To assess the cell bio-behaviors in the intervention of Arg-G4-based PHD2 silencing, the real-time parameters of MSC function and morphology were monitored and recorded by the real-time cell monitoring system, using a Cell-IQ cell culturing platform (Chip-Man Technologies, Tampere, Finland), equipped with a phase-contrast microscope (Nikon CFI Achromat phase contrast objective with 10× magnification) and a camera (Nikon Corporation, Tokyo, Japan).^23^ On 6-well plates, MSCs were transfected with Arg-G4-siRNA complexes using optimized N/P ratio and siRNA concentration. After 6 hours of incubation, the media were exchanged and allowed to continued incubation for 42 hours. Images were captured at 5-minute intervals during the 48 hours. Analysis was carried out with a freely distributed image software (Cell-IQ Imagen v2.9.5c; McMaster Biophotonics Facility, Hamilton, ON, Canada), using the Manual Tracking plugin created by Fabrice Cordelieres (Institut Curie, Orsay, France).

### Optimized gene silencing assay

Using optimized N/P ratio and siRNA concentration, MSCs were transfected by Arg-G4-siRNA complexes as described above. The fluorescence of transfected siRNA could be observed directly under fluorescence microscope (LEICA DM IRE2; Leica, Solms, Germany). PHD2 gene silencing assay was determined by real-time polymerase chain reaction (PCR). Non-transfected MSCs were used as the control. The results were normalized to 18S expression.

### Myocardial infarction models and cell transplantation

MI models were induced on 8-week-old female C57/BL6 mice as described earlier.^24^ Briefly, mice were anesthetized by intraperitoneal administration with a mixture of ketamine hydrochloride (40 mg/kg) and diazepam (5 mg/kg). Mice were endotracheally intubated and mechanically ventilated on a small animal ventilator (KDS model 35; KD Scientific, Holliston, MA, USA) with supplemental oxygen. The heart was exposed through a 2 cm incision at the left lateral costal rib. The left anterior descending coronary artery was permanently ligated with an 8-0 silk suture (Ethicon Inc., Somerville, NJ, USA). Ischemia was confirmed by visual inspection of blanching in the myocardium distal to the site of occlusion (Figure S1).

One day before transplantation, MSCs were transfected with Arg-G4-siRNA complexes. At 1 hour after MI, 50 μL of PBS (Group 1) without MSCs, or with 1×10^6^ male non-transfected MSCs (Group 2), or with 1×10^6^ Arg-G4-siRNA transfected male MSCs (Group 3) were intramyocardially injected at the border zone of the infarct. We used cyclosporine A daily via the intramuscular injection route (10 mg/kg/day) 3 days before transplantation until sacrifice.

### Echocardiography

Echocardiography was carried out on test animals under light sedation using 2% isoflurane in oxygen at 1 day (baseline) and 28 days after MI (n=8 for each studied group). An investigator blinded to the therapeutic intervention on animals performed the echocardiography on a digital ultrasound system (Vevo770; VisualSonics, Toronto, ON, Canada). Left ventricular internal diameters at end-diastole (LVIDed) and left ventricular internal end-systole (LVIDes) were acquired from M-mode echocardiogram. Left ventricular ejection fraction (LVEF) and left ventricular fractional shortening (LVFS) were computed as LVEF =1− (LVIDes/LVIDed)^2^ and LVFS =1− (LVIDes/LVIDed).

### Survival of grafted MSCs

At 2 hours, and then 1, 3, and 7 days after cell transplantation, hearts were harvested from Groups 2 and 3 (n=5 for each group). The whole DNA from homogenized heart tissue was extracted by the DNeasy kit (Qiagen, Dusseldorf, Germany) according to the manufacture’s protocol. The sex-determining region of the Y-chromosome (sry) gene plasmids were generated from male C57/BL6 MSCs; and the standard curves were established by serially diluting the sry plasmids. Real-time PCR analysis was then performed to qualify the sry gene copy number in every harvested heart using the SYBR Green kit (Qiagen).^24^

### IGF-1 expression in myocardium

At 7 days after MI, hearts (n=5 in each group) were harvested and homogenized in 1× TE (Tris-EDTA) buffer containing protease inhibitors. The supernatant was used to determine IGF-1 level in myocardium by ELISA (enzyme-linked immunosorbent assay) kit (R&D Systems, Inc., Minneapolis, MN, USA).

### Histological study

One day after cell transplantation, hearts (n=3 for each studied group) were harvested for frozen section. Terminal deoxynucleotidyl transferase dUTP nick end labeling (TUNEL) detection kit-TMR (Hoffman-La Roche Ltd., Basel, Switzerland) was used to detect apoptotic cardiomyocytes at the peri-infarcted zone according to the manufacturer’s protocol. For the apoptotic index, tissue sections were counterstained with 4′,6-diamidino-2-phenylindole (DAPI; Beyotime, Haimen, People’s Republic of China) after TUNEL. Under fluorescence microscope, total number of cell nuclei (DAPI labeled) and apoptotic nuclei (TUNEL labeled) were counted in five fields for each slide, and the apoptotic index was calculated as the percentage of TUNEL positive nuclei to the total nuclei.

At 28 days after MI, hearts (n=5 in each group) were harvested for Masson trichrome and immunostaining. Under a microscope (Olympus Corporation), scar size and interstitial fibrosis was evaluated in left ventricle. The quantification was calculated using Image-Pro Plus 6.0 (Media Cybernetics, Silver Spring, MD, USA).^24^ The capillary and arteriolar densities at the peri-infarcted zone were measured by staining for PECAM-1 and alpha-smooth muscle actin (α-SMA) (Santa Cruz Biotechnology) (n=5 for each studied group). Blood vessel densities were calculated for five randomly selected high magnification fields in each section. Averages based on five fields from each of the three samples per animal were used for comparison.

### Statistics

The data were analyzed with SPSS 17.0 software (SPSS Inc., Chicago, IL, USA). All values were expressed as the mean ± standard error of the mean. One-way analysis of variance with the post hoc Bonferroni test was performed to assess the significant difference among multiple groups. The significant difference between two groups was evaluated using the Student’s *t*-test.

## Results

### Characterizations of Arg-G4-siRNA complexes

**Figure 2:**
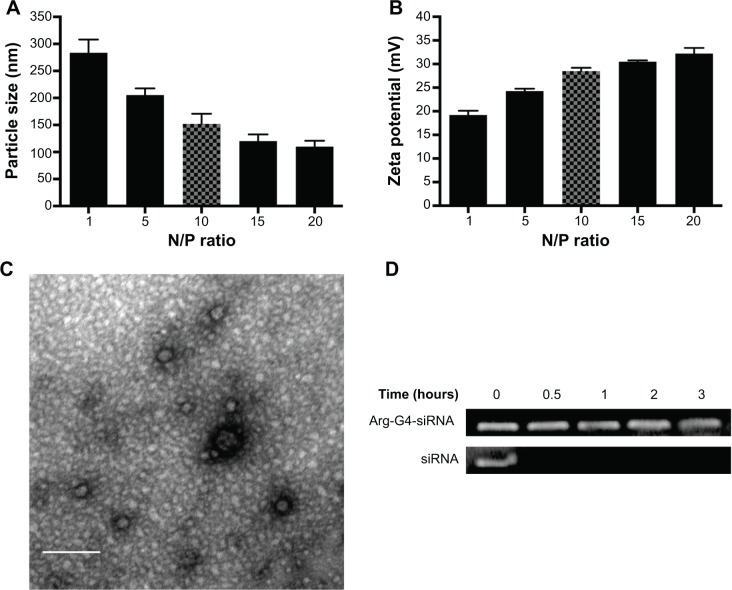
Characterizations of Arg-G4-siRNA complexes. **Notes:** (**A**) Particle size at various N/P ratios. (**B**) Zeta potential at various N/P ratios. (**C**) Transmission electron microscopy image of Arg-G4-siRNA complexes at N/P ratio of 10. (**D**) Arg-G4 encapsulated and naked siRNA against RNase A digestion. **Abbreviations:** Arg-G4, arginine-terminated G4; G4, generation 4 poly(amidoamine); N/P, nanoparticle to DNA nitrogen-phosphorus; siRNA, small interfering RNA.

Particle size and zeta potential of Arg-G4-siRNA complexes at various N/P ratios were shown in Figure 2A and B. At N/P ratio of 10, Arg-G4-siRNA complexes existed as nanoparticles with size of 152.2±18.5 nm. Zeta potential measurement affirmed that the complexes exhibited positive zeta potential values around 28.5±0.7 mV. The morphologies of Arg-G4-siRNA complexes at N/P ratio of 10 are shown in Figure 2C.

As shown in Figure 2D, free siRNA band disappeared after 0.5 hour, suggesting that naked siRNA was prone to be degraded by RNase A. In contrast, siRNA was protected well by Arg-G4 nanoparticles, and remained relatively intact in RNase A even at 3 hours.

### Optimization of Arg-G4-based gene silencing system

**Figure 3:**
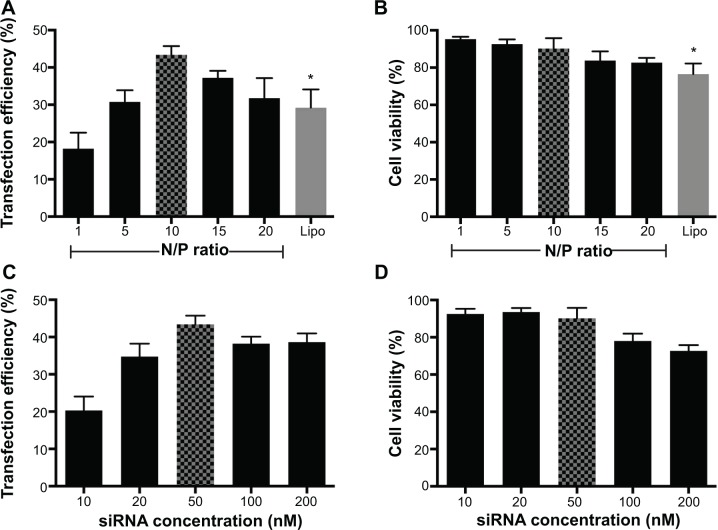
Optimization of Arg-G4-based gene silencing. **Notes:** (**A**) Arg-G4-siRNA transfection efficiency in MSCs at various N/P ratios using Lipofectamine 2000 as a control. (**B**) Cell viability in MSCs during transfection at various N/P ratios using Lipofectamine 2000 as a control. (**C**) Arg-G4-siRNA transfection efficiency in MSCs using various siRNA concentrations. (**D**) Cell viability in MSCs during transfection using various siRNA concentrations. *P<0.05 versus transfection efficiency or cell viability at N/P ratio of 10. **Abbreviations:** Arg-G4, arginine-terminated G4; G4, generation 4 poly(amidoamine); Lipo, Lipofectamine 2000; MSC, mesenchymal stem cell; N/P, nanoparticle to DNA nitrogen-phosphorus; siRNA, small interfering RNA.

Under optimized transfection conditions (N/P ratio =10; siRNA =50 nM), maximum transfection efficiency (43.38%±2.35%) could be achieved with low cytotoxicity (cell viability =90.19%±5.58%). Arg-G4 exhibited greater transfection efficiency and biocompatibility than Lipofectamine 2000 (transfection efficiency 29.23%±4.89%; cell viability 76.57%±5.61%) (Figure 3A and B) (*P*<0.05); and 50 nM was considered as the optimized siRNA concentration during gene silencing (Figure 3C and D).

### Assessment of the optimized gene silencing system

**Figure 4:**
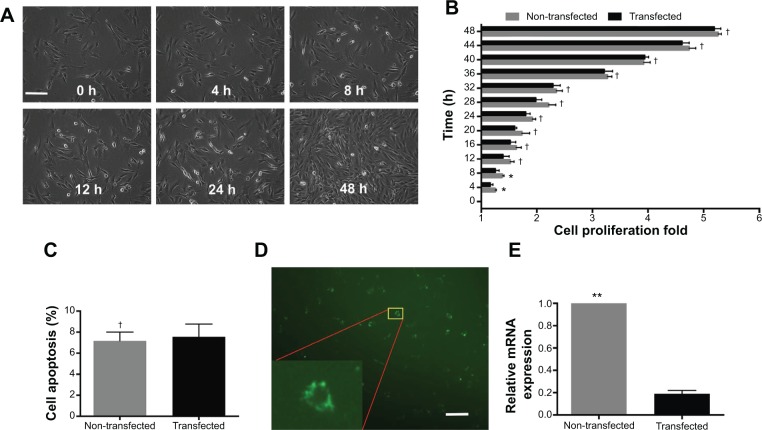
Assessment of the optimized gene silencing system. **Notes: (A)** Real-time MSCs bio-behaviors during transfection. (**B**) Cell proliferation of non-transfected and transfected MSCs during 48 hours after transfection. (**C**) Cell apoptosis of non-transfected and transfected MSCs at 48 hours after transfection. (**D**) FAM-tagged siRNA transfected into MSCs. Scale bar =50 μm. (**E**) PHD2 mRNA expression after siRNA transfection. ^†^*P*>0.05 versus non-transfected MSCs; *P<0.05, **P<0.01 versus non-transfected MSCs. **Abbreviations:** FAM, carboxyfluorescein; h, hour; mRNA, messenger RNA; MSC, mesenchymal stem cell; PHD2, prolyl hydroxylase domain protein 2; siRNA, small interfering RNA.

Under optimized conditions (N/P ratio =10; siRNA =50 nM), the gene silencing system was used to deliver PHD2 siRNA. The real-time cell bio-behaviors were observed by Cell IQ system (Figure 4A) (non-transfected, Video S1; transfected: Video S2). In the first 8 hours, transfected MSCs exhibited a slower proliferation rate than the non-transfected group (*P*<0.05). However, there was no obvious difference of cell proliferation between the transfected and non-transfected MSCs in the remaining 40 hours (*P*>0.05) (Figure 4B). We confirmed that MSCs exhibited low apoptosis at 48 hours after transfection with the gene silencing system (Figure 4C). After FAM-tagged siRNA was delivered by Arg-G4 nanoparticles, fluorescence could be detected in the cytoplasm (Figure 4D). As a result, PHD2 mRNA was downregulated in the MSCs treated with Arg-G4-siRNA, indicating the remarkable downregulation capability of targeting the *PHD2* gene by Arg-G4-siRNA (Figure 4E).

### MSCs survival and IGF-1 level in the myocardium

**Figure 5:**
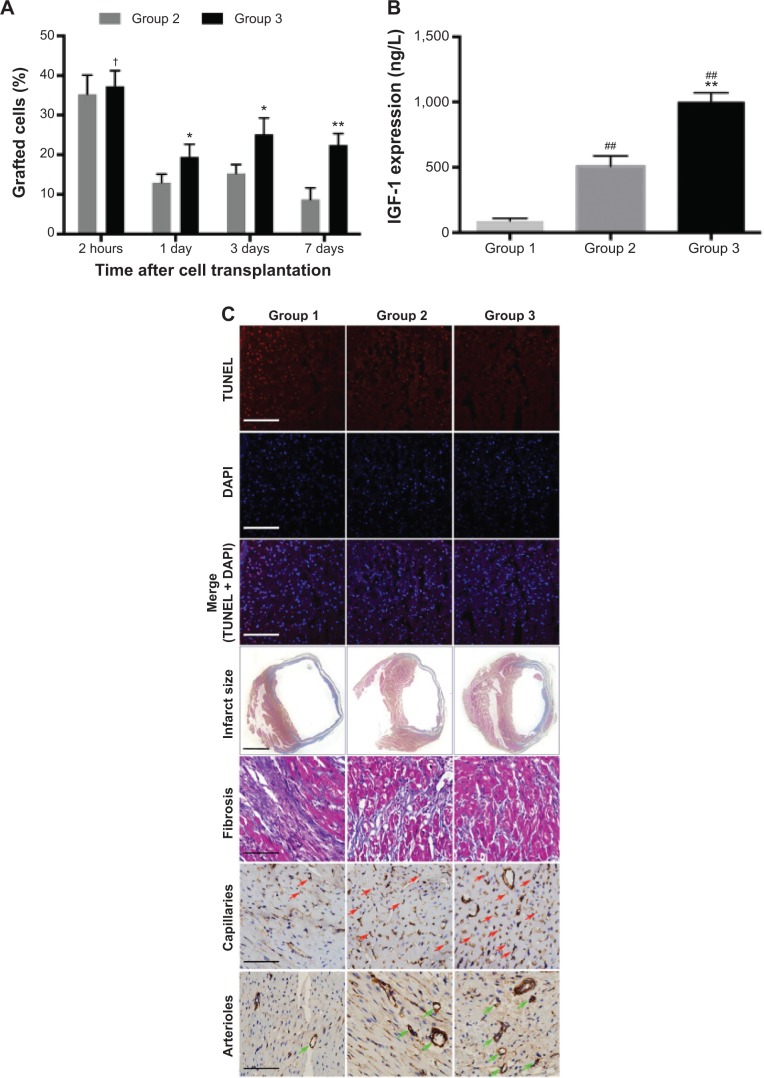

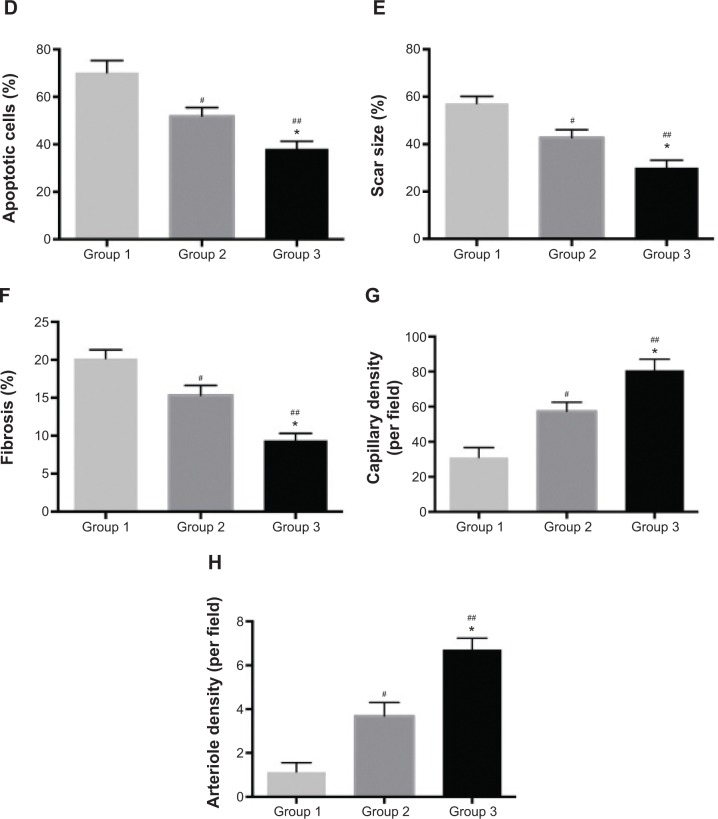
In vivo measurement of Arg-G4-siRNA transfected MSC transplantation. **Notes: (A)** Percentage of graft MSCs in Groups 2 and 3 at 2 hours, and 1, 3, and 7 days after transplantation. (**B**) IGF-1 expression in myocardium. (**C**) TUNEL-labeled apoptotic nuclei and DAPI-labeled myocardial nuclei at the peri-infarcted zone (red, apoptotic nuclei; blue, all myocardial nuclei; purple, merge vision of apoptotic nuclei) (scale bar =100 μm); infarct size (scale bar =1 mm) and interstitial fibrosis (scale bar =50 μm) determined by Masson trichrome; capillaries (brown color, red arrow) and arterioles (brown color, green arrow) at the peri-infarcted zone (scale bar =50 μm). (**D**) Apoptotic cell percentage at the peri-infarcted zone. (**E**) Scar size at 28 days after MI. (**F**) Interstitial fibrosis of left ventricle. (**G**) Capillary density based on PECAM-1 staining. (**H**) Arteriole density based on α-SMA staining. ^†^*P*>0.05 versus Group 2; *P<0.05 versus Group 2; **P<0.01 versus Group 2; ^#^*P*<0.05 versus Group 1; ^##^*P*<0.01 versus Group 1. **Abbreviations:** α-SMA, alpha-smooth muscle actin; Arg-G4, arginine-terminated G4; DAPI, 4′,6-diamidino-2-phenylindole; G4, generation 4 poly(amidoamine); IGF-1, insulin-like growth factor-1; MI, myocardial infarction; MSC, mesenchymal stem cell; siRNA, small interfering RNA; TUNEL, terminal deoxynucleotidyl transferase dUTP nick end labeling.

The survival rate of grafted MSCs in Group 3 was significantly higher than that in Group 2 (2 hours, 37.28%±3.96% versus [vs] 35.27%±4.83%, *P*>0.05; 1 day, 19.48%±3.17% vs 12.97%±2.12%, *P*<0.05; 3 days, 25.19%±4.09% vs 15.32%±2.18%, *P*<0.05; and 7 days, 22.46%±2.89% vs 8.72%±2.91%, *P*<0.01) (Figure 5A). IGF-1 expression in myocardium was significantly upregulated in Group 3 (1,002.46±68.54 ng/L) when compared with Group 1 (88.97±19.87 ng/L) and Group 2 (508.32±78.77 ng/L) (*P*<0.01) (Figure 5B).

### Histology

At 1 day after MI, the apoptotic cardiomyocytes in the peri-infarcted area were stained with TUNEL (Figure 5C). Group 3 exhibited less apoptotic cells (38.18%±3.13%) than Group 1 (70.23%±5.09%) (*P*<0.01) and Group 2 (51.89%±3.62%) (*P*<0.05) (Figure 5D). Four weeks later, left ventricular fibrosis was stained by Masson trichrome staining (Figure 5C). Quantitative analysis revealed that scar size and fibrosis was significantly decreased in Group 3 (30.12%±3.13%; 9.39%±0.92%) compared with Group 1 (57.23%±2.91%, 20.19%±1.14%) (*P*<0.01) and Group 2 (42.79%±3.29%, 15.32%±1.29%) (*P*<0.05) (Figure 5E and F). As shown in Figure 5C, capillaries and arterioles were stained with PECAM-1 and α-SMA, respectively. In Group 3, we found more capillary (80.83±6.23 per field) and arteriole (6.72±0.52 per field) counts at the peri-infarcted zone than those in Group 1 (31.12±5.58 per field and 1.13±0.42 per field) (*P*<0.01) and Group 2 (57.43±5.13 per field and 3.69±0.61 per field) (*P*<0.05) (Figure 5G and H).

### Heart function

**Figure 6:**
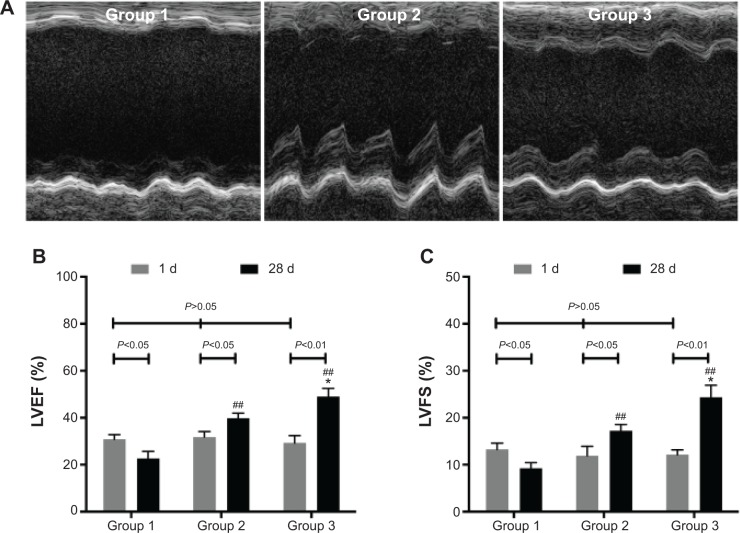
Heart function assessment. **Notes:** (**A**) M-mode echocardiogram of three groups at 28 days after MI. (**B**) LVEF of three groups at 1 day and at 28 days after MI. (**C**) LVFS of three groups at 1 day and at 28 days after MI. *P<0.05 versus Group 2; ^##^*P*<0.01 versus Group 1. **Abbreviations:** d, day; LVEF, left ventricular ejection fraction; LVFS, left ventricular fractional shortening; MI, myocardial infarction.

One day after MI, echocardiography demonstrated the similar LVEF and LVFS to baseline (*P*>0.05). Compared with baseline, Group 1 showed deteriorated LVEF (22.72%±3.02%) and LVFS (9.26%±1.21%) (*P*<0.05) after 4 weeks. However, LVEF and LVFS were improved in Group 2 (39.77%±2.21%, 17.24%±1.34%) (*P*<0.05) and Group 3 (48.98%±3.61%, 24.38%±2.54%) (*P*<0.01). Furthermore, LVEF and LVFS in Group 3 showed greater enhancement than those in Group 2 (*P*<0.05) (Figure 6).

## Discussion

Low survival rate of grafted stem cells limits their therapeutic effect in ischemic myocardium. The PHD2 silencing in stem cells before transplantation is an effective approach to solving the problem. An effective and biocompatible PHD2 siRNA delivery system is quite necessary for clinical application. In the present study, we developed Arg-G4 nanoparticles as a novel siRNA delivery system to silence PHD2 in MSCs. After transplantation of PHD2 silenced MSCs, we observed enhanced survival of grafted cells in ischemic myocardium, which promoted cardiac repair successfully.

Arg-G4 nanovector-based siRNA loading was extremely simple and effective. In the present study, siRNA was mixed directly with Arg-G4 nanoparticles at room temperature. The superior binding ability of Arg-G4 toward siRNA can be explained by the simultaneous presence of positive charges from the primary amine and the guanidine group from PAMAM and arginine residue, which permits a strong and effective interaction with negative charges from siRNA.^18^ After commixture, Arg-G4-siRNA complexes existed as uniform nanoparticles with an average particle size of around 152 nm and positive zeta potential values of around 28 mV, suggesting the sustained colloidal stability of the Arg-G4-siRNA complexes. Furthermore, siRNA could be encapsulated by Arg-G4 nanoparticles and kept from RNase A digestion for up to 120 minutes. The protection effect can maintain siRNA intact during the transfection to insure the gene silencing effect.^25^

High transfection efficiency is necessary for a satisfied gene silencing system. In terms of in vitro transfection, primary stem cells are usually more difficult to transfect than the immortalized cancer cell lines.^26^ Even Lipofectamine 2000, a popular and efficient transfection reagent, cannot transfect gene/siRNA into MSCs with a satisfactory efficiency.^27^,^28^ At N/P ratio of 10, Arg-G4 nanoparticle could transfect siRNA with efficiency of 43.38%±2.35%, which was much higher than that of Lipofectamine 2000. And in vitro study exhibited significant PHD2 gene silencing in MSCs after transfection. The high transfection efficiency was related to the positive zeta potential (28.5±0.7 mV) and suitable particle size (152.2±18.5 nm) of Arg-G4-siRNA complexes, which can favorably interact with the negatively charged cell membrane-associated proteoglycans, thus intensifying membrane penetration. After cell uptake, we supposed that the large number of positive charges within Arg-G4 nanoparticles could be protonated in acidic endosomes, causing the endosomes to swell and finally burst to release the cargo.^29^,^30^ However, the detailed transfection mechanism needs to be investigated in the further study. It has been reported that the arginine-terminated PAMAM of lower generation from Arg-G1 to Arg-G3 showed weak siRNA delivering activity.^18^ This can be ascribed to their structural features associated with lower generation, which cannot provide the sufficient multivalency and cooperativity required to efficiently interact with the siRNA molecules and fulfill the task of transporting them along the journey to the desired site for gene silencing.^18^,^31^ Therefore, Arg-G4 nanoparticles could deliver gene/siRNA into primary MSCs with high transfection efficiency; and an Arg-G4 nanoparticle-based gene delivery system could be used in other primary cells in the future study.

Biocompatible property of any new biomaterial is a serious concern requiring rigorous assessment for the biomedical application.^32^ In our present study, MTT results demonstrated that cell viability remained above 80% when using various N/P ratios during transfection. Cell IQ live cell platform can provide us with the detailed and accurate real-time morphological data of cell bio-behavior after transfection.^23^ After 48 hours of consecutive observation, Arg-G4-siRNA transfected MSCs showed low cell apoptosis. Though the proliferation rate was inhibited in the first 8 hours after transfection, no obvious difference was observed in the remaining 40 hours between transfected and non-transfected cells. We suggested that the initial inhibition appearance might be related to the stress response to the nanoparticle-siRNA complexes. The response could be transient and not harmful for cells. Hence, we confirmed that the Arg-G4 nanoparticle-based gene silencing system exhibited extremely low cytotoxicity in MSCs.

PHD2 silencing reduced grafted cell death via a HIF-1α dependent pathway, and PHD2 silencing in grafted cells limited cardiomyocyte apoptosis through a protective paracrine mechanism mediated by enhanced IGF-1 secretion after the activation of NF-κB signaling.^11^ In the present study, PHD2 silencing in MSCs was performed using the Arg-G4 nanovector. After transplantation, Group 3 exhibited higher MSC survival rate than Group 2 at any time point from 2 hours to 7 days. Besides that, we also found that IGF-1, a protective cytokine for grafted cells and ischemic cardiomyocytes, was significantly upregulated in Group 3. As a result, in vivo study demonstrated decreased cardiomyocyte apoptosis, infarcted scar size and left ventricular interstitial fibrosis, and increased capillaries and arterioles, which could be beneficial for the inhibition of ventricular remodeling and improvement of heart function. These results were consistent with published studies where PHD2 was silenced by lentivirus.^10^,^11^ Therefore, we believed that Arg-G4 nanoparticles, as an alternative to virus vector, could mediate PHD2 silencing successfully and enhance the efficacy of MSC transplantation in vivo. In addition, as a ubiquitously cellular oxygen sensor, the regulatory effects of PHD2 on HIF-1 and NF-κB have been reported in many stem cell types.^8^,^33^ Therefore, it indicated that the Arg-G4-based PHD2 silencing system could be also applied in other therapeutic stem cells, such as adipose-derived stem cells. Collectively, a novel nanovector-based PHD2 silencing system could be effective in MSC-based cardiac repair.

In conclusion, this study showed that Arg-G4 nanovector-based siRNA delivery was effective and biocompatible. The Arg-G4-mediated PHD2 silencing system in grafted MSCs could significantly enhance their survival after transplantation, which benefited for cardiac repair in ischemic myocardium. These data are very promising, and efforts to further apply the nanovector on other gene silencing systems in the clinic are in progress.

## Supplementary material

Figure S1 Construction of MI models on C57/BL6 mice.
**Notes:** (**A**) Exposure of LAD (green arrow) through a 2 cm incision at the left lateral costal rib. (**B**) Permanent ligation of LAD (green arrow) with an 8-0 silk suture. (**C**) Ischemia was confirmed by visual inspection of blanching in the myocardium distal to the site of occlusion (green circle).
**Abbreviations:** LAD, left anterior descending coronary artery; MI, myocardial infarction.

Video S1

Video S2
