# Supplementary figures and images for: Nanovector-based prolyl hydroxylase domain 2 silencing system enhances the efficiency of stem cell transplantation for infarcted myocardium repair
Source: Int J Nanomedicine. 2014 Nov 11;9:5203–15. doi: 10.2147/IJN.S71586 (PMC4243506; doi:10.2147/IJN.S71586)

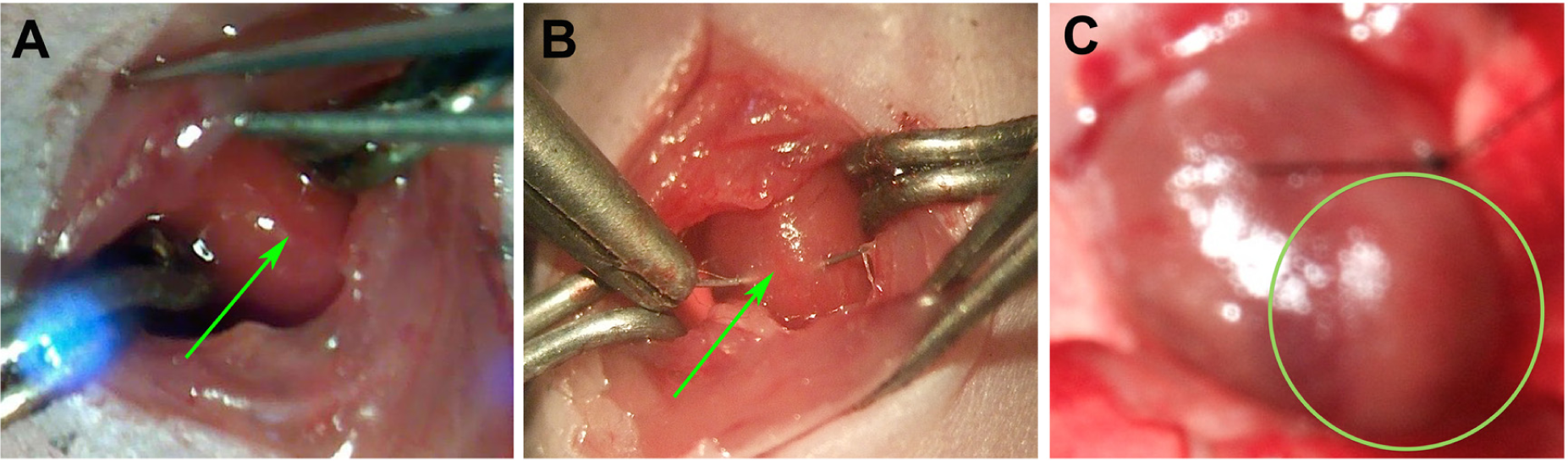

Supplement: Figure S1 — Construction of MI models on C57/BL6 mice. Notes: (A) Exposure of LAD (green arrow) through a 2 cm incision at the left lateral costal rib. (B) Permanent ligation of LAD (green arrow) with an 8-0 silk suture. (C) Ischemia was confirmed by visual inspection of blanching in the myocardium distal to the site of occlusion (green circle). Abbreviations: LAD, left anterior descending coronary artery; MI, myocardial infarction. [file ijn-9-5203s1.tif]
